# Mesenchymal stem cell therapy for liver disease: full of chances and challenges

**DOI:** 10.1186/s13578-020-00480-6

**Published:** 2020-10-27

**Authors:** Xue Yang, Yan Meng, Zhipeng Han, Fei Ye, Lixin Wei, Chen Zong

**Affiliations:** Tumor Immunology and Gene Therapy Center, Shanghai Eastern Hepatobiliary Surgery Hospital, 225 Changhai Road, Shanghai, 200438 China

**Keywords:** Mesenchymal stem cells, Liver disease, Cell therapy

## Abstract

Liver disease is a major health problem that endangers human health worldwide. Currently, whole organ allograft transplantation is the gold standard for the treatment of end-stage liver disease. A shortage of suitable organs, high costs and surgical complications limit the application of liver transplantation. Mesenchymal stem cell therapy has been considered as a promising alternative approach for end-stage liver disease. Some clinical trials have confirmed the effectiveness of MSC therapy for liver disease, but its application has not been promoted and approved. There are still many issues that should be solved prior to using MSC therapy in clinical applications. The types of liver disease that are most suitable for MSC application should be determined, and the preparation and engraftment of MSCs should be standardized. These may be bottlenecks that limit the use of MSCs. We investigated 22 completed and several ongoing clinical trials to discuss these questions from a clinical perspective. We also discussed the important mechanisms by which MSCs play a therapeutic role in liver disease. Finally, we also proposed novel prospective approaches that can improve the therapeutic effect of MSCs.

## Background

With high morbidity and mortality, liver disease presents a major threat to human health. Many stimuli, such as viral hepatitis, alcohol abuse, drugs, metabolic diseases, and autoimmune attack, can trigger chronic/acute liver injury and inflammation, which result in liver failure, cirrhosis and associated hepatocellular carcinoma. Orthotopic liver transplantation is the only effective treatment for liver cirrhosis and liver failure. However, the number of suitable donor organs is very limited. Adults on the waiting list for liver transplantation suffer from a mortality rate of almost 11% [1]. Patients are too weak to wait for suitable donor organs, so the best opportunity for treatment is missed. In addition, liver transplantation is expensive and not available for all patients. There is an urgent need to search for a more effective and feasible treatment for patients with liver cirrhosis and liver failure.

**Fig. 1:**
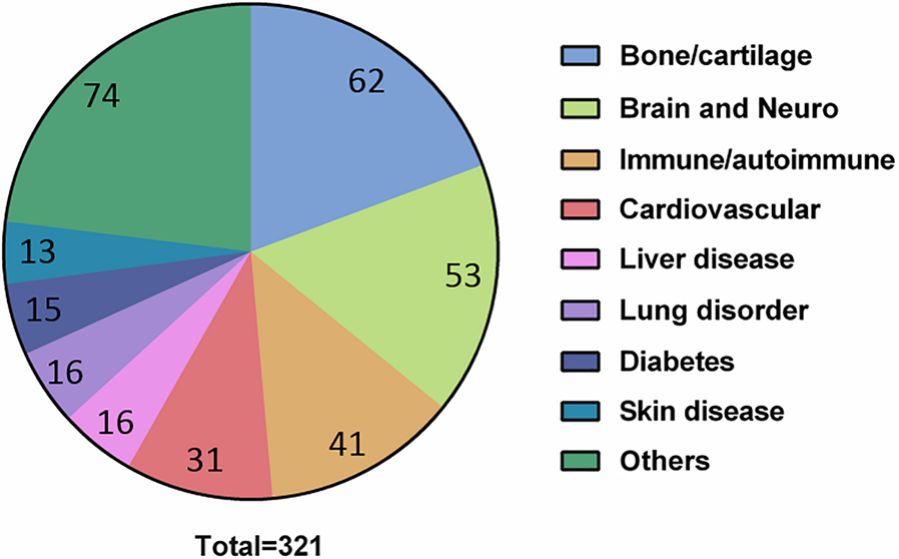
Completed clinical trials based on MSC therapy by disease classification (n = 321)

At present, cell therapy with hepatocytes, hemopoietic cells, immune cells, endothelial progenitor cells and mesenchymal stem cells has been suggested to be a promising candidate therapy for liver diseases [2]. A large body of studies investigated their advantages and disadvantages (Table 1). Among the cell types, MSCs have been the most promising cells because of their many advantages. (1) MSCs can be isolated easily from a wide variety of tissues and can be expanded in vitro without changing their properties. (2) MSCs can be injected into patients by allogeneic transplantation because of their low immunogenicity. Therefore, we can generate reserves of MSCs that we can give to patients at any time. (3) MSCs have the properties of self-renewal and can differentiate into multiple cell types. (4) Researchers have also shown the robust immunomodulation of MSCs. (5) MSCs can also produce secretomes, including soluble factors and exosomes, which can favor regeneration and injury repair. (6) Most importantly, MSCs can migrate to injury sites where they can exert protective effects. Therefore, MSCs have been used to treat various tissue injuries and immune-related diseases in clinical trials. To date, 321 completed clinical trials using MSCs were summarized in Fig. 1 according to the website https://clinicaltrials.gov/. Among them, bone/cartilage, brain/Nero and immune-related diseases account for almost 50% of all MSC-based clinical trials. In addition, we have noticed that 16 clinical trials are related to liver-related diseases. For liver diseases, however, both tissue damage and overactivation of inflammation always go hand in hand. Therefore, from every perspective, MSCs would be the best candidate for cell therapy of liver diseases. However, there are still many issues, and any confusion should be resolved before MSC application in the clinic.

**Table1:** Advantages and disadvantages among different cell types for the treatment of liver diseases

Cell types	Advantages	Disadvantages	References
Hepatocytes	Suitable for many enzyme deficiency states, metabolic diseases, coagulation disorders, as well as liver failure Key metabolic and synthetic cell	Donor organ shortages Limitations in transplanted cell engraftment and proliferation Susceptible to infection with hepatitis viruses	[104]
Hemopoietic cells	Plasticity is not limited to the tissue they derived from	Procedure for obtaining (bone marrow aspirate)	[105, 106]
Immune cells	Easy to isolate and expand Autologous therapy	Only used in HCC Tend to form inflammatory storms	[107]
EPCs	Appear anti-fibrotic and pro-regenerative Autologous therapy	Isolation process is complicated Clinical use unclear	[108, 109]
MSCs	Easy to isolate and expand Multiple differentiation Generally immune tolerance Could be used with other cell types to reduce inflammation Secretome can be used Both autologous and allogeneic therapy	Some clinical studies have been negative Poorly defined cell type	[5, 110–112]

In this review, several issues that must be addressed during clinical treatment of MSCs are discussed from clinical perspectives. (1) Which kind of liver disease is most suitable for MSC application? (2) How should we choose the best source of MSCs, best doses of MSCs and best engraftment route of MSCs? (3) What are the mechanisms by which MSCs play a therapeutic role in liver disease? (4) Finally, we propose potential approaches to enhance the therapeutic effect of MSCs, including the use of modified MSCs, pretreated MSCs and cell-free therapy with MSCs. To address these issues, we examined both ongoing and published clinical trials of MSC applications in liver diseases. Some preclinical tests were also used to assist in drawing a conclusion. We would like to clear up any confusion about MSC applications in liver diseases and help patients receive more appropriate treatment. This review is intended to be beneficial for creating a new strategy for MSC application and enhancing the therapeutic effect of MSCs. It will also help to develop a more specific standard for MSC application.

## Which kind of liver disease is most suitable for MSC application in the clinic?

MSCs have been shown to exert beneficial effects in a range of clinical settings, including the treatment of degenerative and immune-mediated diseases while also being reported to ameliorate liver injury in the setting of both acute and chronic liver damage. However, whether MSC-based therapy is more effective than conventional treatment and which type of liver disease is best suitable for MSCs application in clinic remained unclear.

**Fig. 2:**
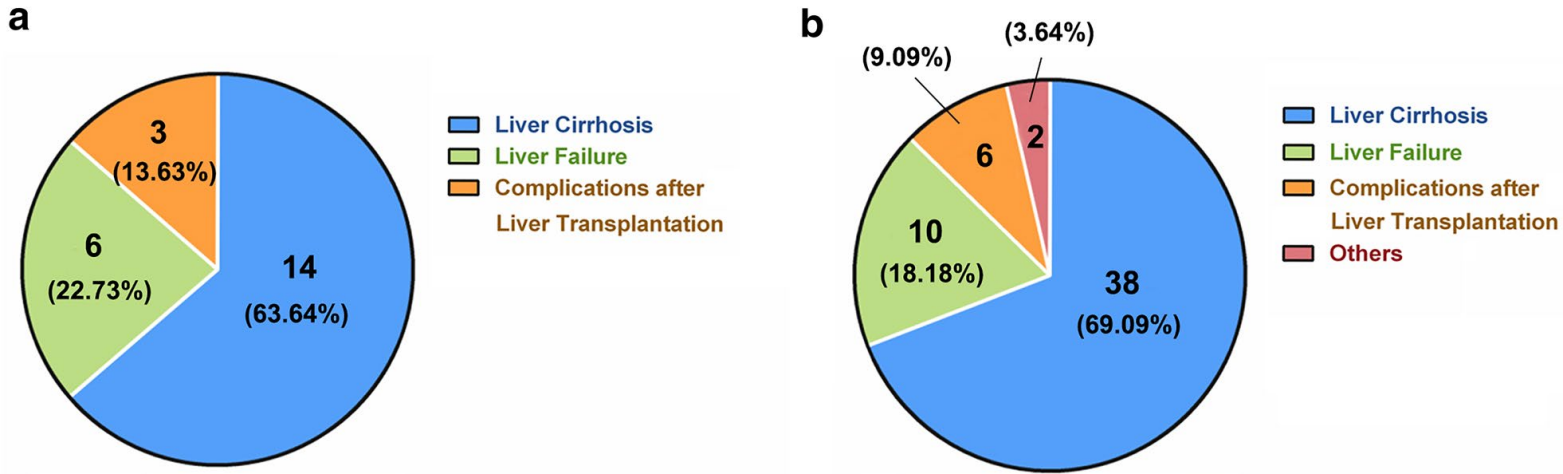
The number and percentage of MSC based completed (**a**) and ongoing (**b**) clinical trials classified by liver disease type

To find out the indications of MSCs in the treatment of liver disease. In this review, we collected a total of 22 clinical trial articles on MSCs treatment for liver disease published from 2007 to 2018 (Table 2). As shown in the pie chart in Fig. 2a, 14 of them are about MSCs therapy for liver cirrhosis. The other five articles are related to liver failure, and three of them are related to complications after liver transplantation. We found that most of these diseases have a common feature, that is, they are all end-stage liver diseases. And for them, the only effective approach is organ transplantation, but its practical application is constrained by some well-known reasons such as the limited availability of donor organs, surgical complications, immunological side effects, and high medical cost [3]. The development of alternative methods for treatment of liver disease is highly requested. Therefore, stem cell therapy, and regenerative medicine are being investigated to improve the prognosis of patients with those end-stage liver diseases. Evaluation of the end points in these studies revealed the safety and efficacy of human MSC transplantation. While there are some differences in the therapeutic effects of different types of liver diseases.

**Table 2:** Clinical trials using MSCs to treat liver disease

Liver diseases	Type of clinical trials	No. of patient treated	No. of control patient	Source of MSCs	Route	Dose	Relevant indicators and patient response	Refs
Decompensated liver cirrhosis	No control group	4	0	Autologous, bone marrow	Peripheral vein	3 × 10^7^	Slightly improvement of liver function tests and MELD scores in two of four patients	Mohamadnejad et al*.* [113]
Liver cirrhosis	No control group	8	0	Autologous, iliac crest	Peripheral or the portal vein	3–5 × 10^7^	Improvement of liver function and MELD scores	Kharaziha et al*.* [9]
Liver failure caused by hepatitis B	Randomized Controlled trial	53	105	Autologous, bone marrow	Hepatic artery	None	Short-term efficacy was favorable, but long-term outcomes were not markedly improved	Peng et al*.* [14]
Liver failure caused by hepatitis C	Randomized controlled trial	20	20	Autologous, bone marrow-derived hepatocyte	Intrahepatic, intrasplenic	2 × 10^7^ hepatic lineage committed cells	Significant improvement in Child score, MELD, fatigue scale, and performance status	Amer et al*.* [17]
Primary biliary cirrhosis	Non-control	7	0	Allogenic, umbilical cord	Peripheral vein	0.5 × 10^6^ cells/kg body	Decrease in serum alkaline phosphatase and g-glutamyltransferase levels	Wang et al*.* [12]
Acute-on-chronic liver failure	Parallel-controlled trial	24	19	Allogenic, umbilical cord	Cubital vein of the arm	0.5 × 10^6^ cells/kg body	Improvement of the survival rates, MELD and liver function	Shi et al*.* [15]
Decompensated liver cirrhosis	Paired, controlled study	30	15	Allogenic, umbilical cord	Intravenously	0.5 × 10^6^ cells/kg body	Improvement of liver function and reduction in the volume of ascites	Zhang et al*.* [4]
Liver cirrhosis caused by hepatitis C	Randomized controlled trial	15	10	Autologous, bone marrow	Intravenously	1 × 10^6^ cells/kg body	Improvement of liver function, and decline of elevated bilirubin and MELD score	Ansary et al*.* [5]
Decompensated liver cirrhosis	Randomized controlled trial	15	12	Autologous, bone marrow	Peripheral vein	2 × 10^8^	No improvement of Child scores, MELD scores, and liver function	Mohamadnejad et al*.* [8]
Liver cirrhosis caused by hepatitis C	No control group	20	0	Autologous, bone marrow	Intrasplenic	1 × 10^7^	Improvement of liver function	Sabry et al. [46]
Alcoholic cirrhosis	No control group	11	0	Autologous, bone marrow	Hepatic artery	5 × 10^7^	Improvement of Child–Pugh score,and decrease of TGFb1,a-SMA	Jang et al*.* [114]
End-stage liver disease related with HCV	Randomized controlled trial	20	20	Autologous, bone marrow	Peripheral vein	None	54% showed near normalization of liver enzymes and improvement in liver synthetic function	Salama et al*.* [18]
Liver cirrhosis caused by hepatitis B	Randomized controlled trial	20	19	Autologous, bone marrow	Hepatic artery	None	Improvement of liver function and an increased Treg/ Th17 ratio	Xu et al*.* [6]
Liver cirrhosis	No control group	12	0	Autologous, bone marrow	Peripheral vein	1 × 10^6^/kg	Partly improvement of MELD, no change in liver regeneration and fibrosis after 6 months	Kantarcıoğlu et al*.* [115]
Alcoholic cirrhosis	Randomized controlled trial	37	18	Autologous, bone marrow	Hepatic arterial	5 × 10^7^, one-time or two-time	Improvement of liver function and Child–Pugh scores	Suk et al*.* [10]
Acute Liver Allograft Rejection	Randomized controlled trial	14	13	Allogenic, umbilical cord	Intravenously	1 × 10^6^/kg body	Improvement of liver function and allograft histology, increased Treg/ Th17 ratio, CD4 T-cell activation; elevated Levels of TGF-b1 and PGE2	Shi et al*.* [19]
Acute-on-chronic liver failure	Randomized controlled trial	56	54	Allogenic, bone marrow	Intravenously	1.0 to 10 × 10^5^ cells/kg	Improvement of survival rate, liver function, reduction of infection	Lin et al*.* [16]
Liver cirrhosis caused by autoimmune disease	No control group	26	0	Allogenic, umbilical cord, umbilical cord blood, bone marrow	Intravenously	1 × 10^6^/kg	Improvement of liver function and MELD scores	Liang et al*.* [11]
Liver transplantation	Non-randomized, controlled trial	10	10	Allogenic, bone marrow	Intravenously	1.5–3 × 10^6^ /kg	Not sufficient to allow withdrawal of immunosuppression	Detry et al*.* [20]
Liver cirrhosis	No control group	4	0	Autologous, adipose	Hepatic artery	6.6 × 10^5^ cells/kg	Improvement of liver function, and HGF, IL-6 increased	Sakai et al*.* [116]
Ischemic-type biliary lesions following liver transplantation	Randomized controlled trial	12	70	Allogenic, umbilical cord	Peripheral intravenous infusion	1 × 10^6^/kg, 6-time	Improvement of survival rate and liver function	Zhang et al*.* [117]
Liver cirrhosis	Randomized controlled trial	50	53	Allogenic, umbilical cord	Intravenously	(4.0–4.5) × 10^8^	Improvement of Child scores, MELD scores, and liver function	Fang et al*.* [7]

### MSC therapy for liver cirrhosis

Liver cirrhosis (LC) is a complication of liver disease that involves the loss of liver cells and irreversible scarring of the liver. In the 14 articles, patients with cirrhosis caused by many forms of liver disease, such as chronic viral hepatitis (n = 5), chronic alcohol abuse (n = 2), primary biliary cirrhosis (n = 1), autoimmune hepatitis (n = 1) and other conditions (n = 5 heterogeneous cirrhosis), were included. We believe that the experimental results related to LC caused by viral hepatitis are the most convincing because 4 articles contained large samples of data and control groups. In two of the clinical studies mentioned above that were published in 2012, 30 patients with chronic hepatitis B-related LC and 15 patients with chronic hepatitis C-related LC received MSC transfusion. In both studies, compared to the control group, the patients transplanted with MSCs showed significantly improved liver function, as indicated by the elevation of serum albumin levels, a decrease in total serum bilirubin levels, and a decrease in the sodium model for end-stage liver disease score (MELD score) [4, 5]. Consistent with the above study, two other clinical trials related to LC caused by hepatitis B were carried out by different research institutes and clarified that MSC transplantation further improved the liver function, MELD scores and Child–Pugh classification of patients [6, 7]. In the trials conducted by Fang, as many as 103 patients were recruited to participate in the studies; 50 were in the transplant group, and the other patients were in the control group. Controlled trials with larger cohorts of patients have further confirmed the feasibility of MSC transplantation therapy for virus-related cirrhosis.

However, not all studies have revealed MSCs to have the desired treatment effect on liver cirrhosis. Mohamadnejad et al*.* [8] enrolled a total of 27 patients with decompensated cirrhosis and ruled out viral-associated cirrhosis. The patients in this pilot study were heterogeneous regarding the etiology of liver cirrhosis. The results showed that at the 12 months follow-up, the absolute changes in the Child scores, MELD scores, serum albumin, international normalized ratio (INR), serum transaminases and liver volumes did not differ significantly between the MSC and placebo groups. This indicates that based on this randomized controlled trial, autologous bone marrow MSC transplantation through the peripheral vein probably has no beneficial effect in cirrhotic patients. However, based on the above discussion, MSC therapy has a positive effect on patients with viral-associated cirrhosis. Thus, we speculated that there may be a certain correlation between the therapeutic effect of MSCs and the cause of cirrhosis. Interestingly, previous work reported in 2009 by the same author showed that 2 months after MSC injection, all patients had an improved general condition, quality of life and liver function [9]. Why are the results of these two studies completely contradictory? We note that the beneficial effects of MSC transplantation shown in the report published in 2009 are based on the absence of control experiments. Even if we observed improvement, we could not claim that such improvement is definitely related to MSC transplantation. It proved that the controlled trial is critical to the reliability of clinical trial results. Thus, clinical trials associated with heterogeneous LC that lack a control group have not been explored in depth here.

Furthermore, another phase 2 trial with a control group was related to alcoholic cirrhosis. Fifty-five patients (18 in the control group, 18 in the one-time MSC group, and 19 in the two-time MSC group) completed the study. In the fibrosis quantification (before versus after), the one-time and two-time BM-MSC groups were associated with 25% and 37% reductions in the proportion of the collagen area following BM-MSC therapy, respectively. While no significant change in fibrosis quantification was observed in the control group. These results were further confirmed by the Laennec fibrosis score and Child–Pugh score [10]. In summary, judging from existing clinical trial articles, MSC treatment may serve as a potential supplementary therapeutic tool to improve liver function in patients with viral-related cirrhosis and alcoholic cirrhosis.

To date, only a few large controlled trials have been conducted to treat liver cirrhosis patients with MSC transplantation. Several articles related to LC with a small amount of data or a lack of a control trial also showed positive treatment effects for MSCs, which indicated that MSC therapy is also promising for the treatment of cirrhosis induced by other factors, such as autoimmune hepatitis and primary biliary cirrhosis; however, larger samples and double-blind controlled trials are needed for further verification [11, 12].

### MSC therapy for liver failure

Liver failure is a major health problem worldwide due to the variety of acute or chronic injuries that are induced by alcohol consumption, hepatotoxic drugs, autoimmune attack of hepatocytes, or infection with viruses, such as hepatitis B virus (HBV) and hepatitis C virus (HCV) [13]. In China, HBV infection accounts for the highest proportion of liver failure cases. Therefore, Peng et al. [14] enrolled 53 patients with liver failure caused by hepatitis B, who underwent MSC transplantation (105 patients served as the control group). The results showed that MSC transplantation is safe for patients with liver failure caused by chronic hepatitis B. The short-term efficacy was favorable, but long-term outcomes were not markedly improved. This is in agreement with the results of Shi et al. and Lin et al. who reported trials of MSC transfusion for acute-on-chronic liver failure (ACLF) patients in 2012 and 2017, respectively. In both trials, all the patients tolerated the transplantation well until the end of the follow-up period. MSC infusion is convenient for patients with HBV-related ACLF and significantly increases the survival rate by improving liver function and decreasing the incidence of severe infections [15, 16].

In addition to hepatitis B, hepatitis C is also a major form of viral hepatitis that has a very high incidence in Egypt. Therefore, a research institute in Egypt reported a trial that used autologous MSC-derived hepatocyte-like cells for end-stage liver failure therapy. The transplantation group showed improved liver function, including increases in serum albumin, Child–Pugh scores, MELD scores, and performance status, versus the controls [17]. Additionally, for patients with HCV-related end-stage liver disease, another study in Egypt provided evidence that administration of MSCs followed by granulocyte colony stimulating factor (GCSF) mobilization is excellent for liver stem cell therapy to retain liver mass and restore liver functions [18]. In summary, the above large controlled trials demonstrated that MSCs play a supportive role in the treatment of liver failure and show satisfactory tolerability and beneficial effects on liver synthetic functions and hepatic fibrosis resolution.

### MSC therapy for complications of liver transplantation

Orthotopic liver transplantation is the only curative measure for patients with end-stage liver failure. However, the risk of complications after liver transplantation is still high, even with increases in surgical expertise. Two of the most common complications following liver transplant are rejection and infection. MSCs offer new therapeutic opportunities to prevent and treat solid organ transplant rejection. A recent pilot study demonstrated that UC-MSC therapy can alleviate liver damage and improve allograft histology in liver transplant patients with acute graft rejection who did not respond to immunosuppressive agent dose adjustments. However, the study was not carried out long enough to determine whether decreased infection resulted from MSC infusion [19].

However, in the same year, Detry et al*.* [20] enrolled 10 liver transplant patients to receive MSCs infusion and another 10 patients acted as controls. It was shown that no difference in the overall rates of rejection or graft survival was observed between the MSC infusion group and the control group. And Month-6 biopsies did not demonstrate a difference between groups. The study indicated that the immunosuppression weaning in MSC recipients was not successful. The results are completely different and contradictory compared to those of the above pilot study. We analyze that the possible reason might be that the aim and experimental design of the two studies were different. The pilot study aimed to examine the clinical feasibility of MSC infusion as a therapeutic option for liver allograft rejection, and all patients were treated with conventional immunosuppressive agents in both the experimental and control groups, showing the supportive effect of MSC infusion. The study reported by Detry et al. aimed to evaluate the feasibility and tolerability of a single infusion of MSCs in liver transplant recipients, and progressive immunosuppressive withdrawal was attempted in stable patients who received MSCs, which ultimately failed to promote tolerance. Therefore, we speculate that perhaps in the presence of immunosuppressive agents, MSCs can enhance the overall immunosuppressive effect; when the immunosuppressive agents are withdrawn, MSCs cannot serve alone as an alternative treatment. Another reason for this may be that the enrolled patients in the two studies were not truly similar. In the pilot study, the patients were considered suitable if their liver function did not respond to adjustment of immunosuppressive agents. These patients may be more sensitive to MSC application. Therefore, in the clinical application of MSCs, not only the use of an appropriate disease type but also the use of appropriate enrolled patients and a reasonable experimental protocol design should be considered.

Besides, 56 establishment of practical applications of MSCs involve clinical trials to investigate their therapeutic potential for treatment of liver disease, which also almost including decompensated liver cirrhosis, liver failure and complications after liver transplantation, according to ClinicalTrials.gov (Fig. 2b). And Based on the data from the published clinical trials we discussed above, combined with the relevant basic research we have carried out and some of the currently approved MSC-based products for clinical applications, we believe that MSCs are likely to be more effective in the treatment of inflammation-related liver diseases and liver transplant rejection complications.

## Preparation and engraftment of MSCs

### Sources of MSCs

**Fig. 3:**
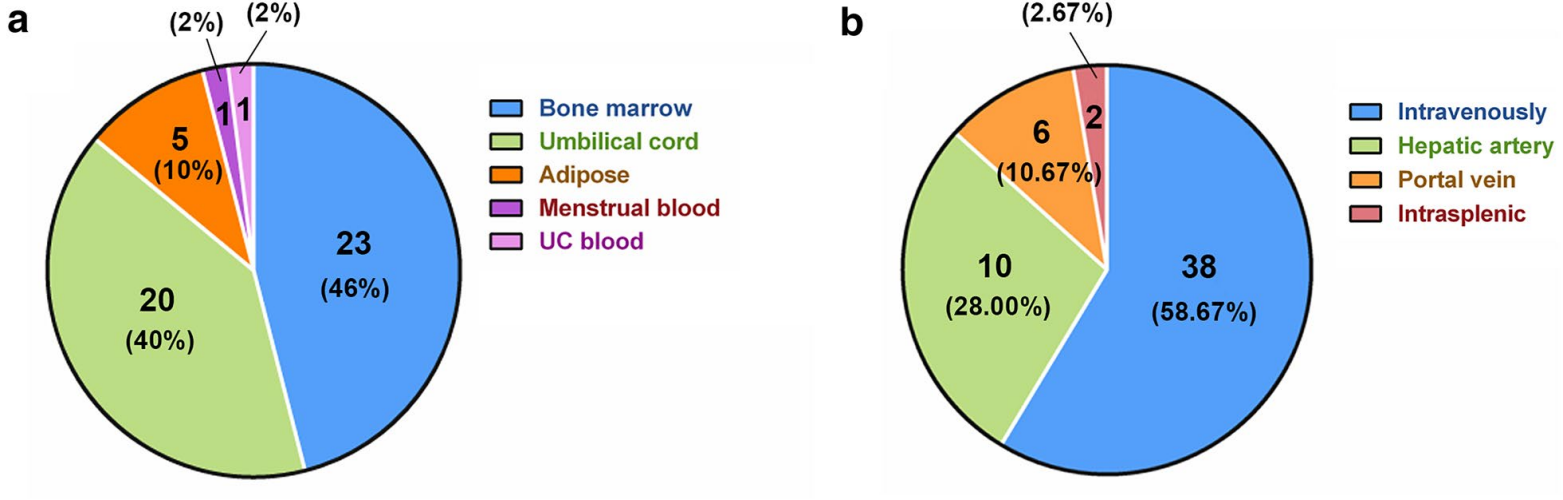
The number and percentage of MSC based clinical trials classified by sources (**a**) and transplantation routes (**b**) of MSCs

Mesenchymal stem cells were first obtained from bone marrow (BM) in 1970 [21], and they are now found to reside in various tissues apart from BM, including adipose tissue [22], umbilical cord (UC) [23], umbilical cord blood [24], placenta [25], amniotic fluid [26], amniotic membrane [27], dental pulp [28], synovium [29], peripheral blood, liver, lung [30], skeletal muscle [31, 32], hair follicles [33] and many others. Currently, MSCs applied for liver disease treatment in the clinic are isolated mostly from the umbilical cord and bone marrow, and in only a few cases adipose and menstrual blood-derived MSCs have been used (Fig. 3a). This is probably because BM-MSCs and UC-MSCs are most thoroughly studied types of cells, and BM and UC are relatively easy to obtain. However, there is still no definite standard for which source of MSCs should be used for clinical application. Many factors should be considered.

BM-MSCs are most suitable for autologous transplantation. However, BM-MSCs have several limitations that should be considered. First, the invasive isolation method is a risk to patients, even to healthy donors, because invasion causes an injury that could lead to inflammation. The proportion of MSCs in bone marrow is not always very high, and they are difficult to proliferate in vitro [34]. In addition, fresh bone marrow from young donors is very limited. Most BM-MSC injections involve autologous transplantation in the clinic. Thus, the condition of patients also limits the number and potential of MSCs. For instance, age-related declines in proliferation and differentiation may weaken the therapeutic effect of MSCs [35].

However, UC-MSCs have many advantages. The isolation process used to obtain MSCs from UC does not cause damage, and it allows for utilization of tissue that might otherwise have been thrown away. It has been reported that the proliferation ability of UC-MSCs is much higher than that of BM-MSCs, [36] which fulfills the need for the large quantities required in the clinic. UC is at an early phase of organic development, so UC-MSCs show higher self-renewal and differentiation capacity [37]. Thus, there is no need to be concerned about age-related issues. On the other hand, UC-MSCs are better for liver failure treatment because of higher differentiation capacity. In addition, it has been demonstrated that the immunogenicity of UC-MSCs is lower than that of BM-MSCs [38, 39]. Thus, for patients with liver transplantation, autologous BM-MSCs and allogenic UC-MSCs are more appropriate. Adipose tissue is also easy to obtain, and the isolation method is very simple. However, AD-MSCs have been shown to have poor proliferation and self-renewal ability. Their anti-inflammatory ability is lower than that of BM-MSCs [40]. In conclusion, UC-MSCs are a prominent and preferred candidate for clinical application.

During clinical treatment, the choice between autologous or allogeneic transplantation is a source of confusion for doctors. For autologous transplantation, the type and amounts of MSC-derived tissues are very limited. Therefore, it will take a long time to obtain a sufficient number of MSCs for transplantation. The wait for cells might cause doctors to miss the window for treatment for liver diseases such as liver failure and acute liver failure. Obviously, the process of obtaining tissue also causes injury to patients. On the other hand, due to their lower immunogenicity, allogeneic MSCs can be injected without rejection risk, and an enough MSCs can be obtained very quickly. We can also choose the MSCs with the best properties. Therefore, allogeneic MSCs may be much better than autologous MSCs.

### Routes of MSC transplantation

In clinical trials, MSCs have been transplanted into patients via various available routes; most are transplanted by intravenous injection, followed by intrahepatic injection (including via the portal vein and hepatic artery), and the least used method is intrasplenic injection (Fig. 3b). Which route is the best and most beneficial for therapy? How can the best route be chosen from all routes? It is indeed necessary to explore this question.

There are only a few completed clinical trials that have used multiple infusion routes simultaneously. In one clinical trial that used BM-MSCs to treat end-stage liver failure that was performed in Egypt, MSCs were injected into patients by the portal vein and intrasplenic injection, and the effects of these two injection routes for 6 months after transplantation were compared [17]. As a result, it was found that portal vein injection was more effective than intrasplenic injection, as indicated by the fatigue impact scale and MELD score. However, this effect was observed only in the first month, and the difference disappeared in the following days. This suggests that portal injection favors faster engraftment of injected cells but does not affect the total number of engrafted cells. Splenic injection is much easier than intrahepatic injection technically. However, we need to consider the complications induced by intrasplenic injection. Seventy percent of patients with intrasplenic injection had fever after cell transplantation, and 30% of patients had fever after intrahepatic injection. Thus, we prefer portal vein injection between these two injection routes.

Apart from portal vein injection, the hepatic artery can be used for intrahepatic injection. Hepatic artery injection is used more often than portal vein injection during MSC transplantation in clinical trials. However, is hepatic artery injection better than portal vein injection? Sang et al. concluded that intraportal injection was better for repairing liver injury in swine with ALF than hepatic intra-arterial injection, peripheral intravenous injection and in situ intrahepatic injection, as indicated by the increase in survival time and the decrease in liver injury [41]. Another study showed that transplantation via the hepatic artery was not beneficial for the transdifferentiation of MSCs.

Intravenous injection is the most popular route for clinical therapy because of its convenience. Is it the optimal method for cell transplantation? We must look for evidence from preclinical trials because there are no clinical trials comparing the effectiveness of intravenous injection and other routes. Animal studies have shown that cells transplanted via intravenous injection will accumulate in the lung. E. Eggenhofer demonstrated that in the first few hours, 60% of MSCs injected intravenously accumulated in the lungs and did not move to the liver afterward. These cells were probably cleared by immune cells [42]. Higashimoto et al*.* [43] showed that, after intravenous injection in mice with ConA induced hepatitis, GFP-labeled MSCs were found only in the lung but not in the liver. Cao et al. [44] determined that intraportal injection of MSCs restored hepatic function in ALF pigs, while transplantation of MSCs via the jugular vein did not. Li et al. [45] also administered MSCs via the peripheral vein and intraportal route into ALF pigs. They reported that the injection of MSCs via a peripheral vein did not rescue ALF pigs, while most of the ALF pigs survived for over 6 months after transplantation of MSCs via intraportal injection.

Intraportal injection is obviously the optimal route for MSC transplantation to treat liver diseases because of faster engraftment and the avoidance of off-target accumulation. In fact, we must evaluate the condition of patients and the potential risk of using a specific route before choosing the injection route. For instance, we need to know if there is thrombosis in the portal vein or technical issues. If there are difficulties involved in intraportal injection, intrasplenic injection or intravenous injection is probably a widely accepted alternative approach, and more MSCs are suggested to be prepared.

### Doses used for MSC transplantation

The doses of MSCs applied in clinical therapy were not exact. It is very difficult to assess an optimal dosing strategy for MSC transplantation because most of the recent clinical trials were aimed at observing the efficiency of MSC therapy but did not determine the optimal dose. Even so, we can still provide some guidance based on these clinical trials. Most clinical trials apply MSCs according to the body weight of patients (n = 9, 0.5–3 × 10^6^/kg as a single dose), while others apply MSCs according to the total quantity of cells (n = 7, 1–20 × 10^7^). According to other medicine dosing strategies, injection according to body weight may be more reasonable. An Egypt study in 2013 involving transplanting MSCs into patients with liver cirrhosis showed that as few as 1 × 10^7^ MSCs were effective for 6 months without any side effects [46]. In the same year, another study used MSCs in patients with liver cirrhosis and found that 2 × 10^8^ MSCs showed no significant effect after 12 months compared with placebo [8]. However, there are other effective clinical trials that used more than 2 × 10^8^ MSCs. It is important to bear in mind that we cannot draw a conclusion from different trials. There are other parameters involved in this process, such as the condition of patients, progression of diseases and treatment regimens. Among the recent clinical trials involving applying MSCs to treat liver diseases, the total number of MSCs used was from ~ 10^7^−  ~ 10^9^, regardless of which method was chosen to deliver the dose. The large range of doses used is hard to explain because there are few studies involving comparisons of different doses in the same clinical trial. However, we know that as few as 1 × 10^7^ cells can be helpful.

Most completed clinical trials injected MSCs only once, and others injected MSCs multiple times, with up to 9 injections in one experiment [*ClinicalTrials.gov* NCT02223897]. How long would one dose of MSCs be effective? Is it necessary to inject MSCs several times? One study that used MSCs for liver cirrhosis treatment showed that MSCs could improve liver function over two years. No difference was observed between the control group and MSC group after two years [17]. As a result, the effect of one injection will not last that long. However, another clinical trial involving treatment for liver cirrhosis showed that there was no significant difference between the effect of one or two injections during a year [10]. This is probably because the second injection was performed only one month after the first injection. From these two studies, we can conclude that a longer interval for the second injection is probably beneficial for improving the therapeutic efficiency of MSCs.

## Mechanism of MSC therapy for liver disease

In order to elucidate how MSCs play a positive role in the treatment of the above-mentioned liver diseases, we present the current findings regarding the molecular mechanism involved in the MSC-dependent modulation of liver diseases via a brief overview. A large number of studies have reported that MSC transplantation could promote partial recovery of liver function and alleviate liver inflammation in several animal models of liver fibrosis or cirrhosis. The mechanisms of the effects of MSCs on treating liver diseases have been evaluated from various perspectives in basic studies.

### Immunomodulation capacity of MSCs

Most previous studies have shown that MSCs could improve or repair injured tissue by modulating tissue immune responses through direct cell-to-cell interaction or paracrine secretion. MSCs could modulate innate and adaptive immune responses.

The induction of CD4^+^CD25^+^FoxP3^+^ regulatory T cells (Tregs) is critical for MSC-mediated immunomodulation. Previous studies indicated that an imbalance in Treg/Th17 cells may be associated with liver diseases, such as autoimmune hepatitis, chronic hepatitis B, and alcoholic liver disease. A random trial reported that compared with those in the control group, the serum levels of interleukin-17 (IL-17), tumor necrosis factor-α (TNF-α), and interleukin-6 (IL-6) were significantly lower in the transplantation group. Furthermore, a significant increase in Tregs and a marked decrease in Th17 cells were observed in the transplantation group, indicating that BM-MSCs exhibit potent immunosuppressive and anti-inflammatory effects through the regulation of serum levels of inflammatory cytokines [6]. In line with the above study, Shi et al*.* [19] reported a pilot study using MSCs for treating liver transplant recipients. The data showed that after MSC infusion, Tregs in the liver were upregulated, whereas Th17 cells were downregulated. They also found that the percentage of HLA-DR^+^ CD4^+^ T cells was significantly decreased after UC-MSC infusions, which may facilitate the suppression of alloreactive responses. To date, only a few clinical protocols have included ex vivo immunologic studies to gain insight into the mechanistic effects of MSC therapy in liver disease. There is an increasingly large body of work that supports the immunoregulatory capacity of transferred MSCs in rodent models of liver disease. In nonclinical experiments, several different molecules secreted by MSCs have been reported to have an immunomodulatory effect on T cell activities, including inducible nitric oxide synthase (iNOS), hepatocyte growth factor (HGF), prostaglandin E2 (PGE2) and transforming growth factor (TGF)-β [47, 48]. Another study demonstrated that MSCs can secrete matrix metalloproteinases (MMPs), such as MMP-2 and MMP-9, that suppress T cell activation by cleaving surface CD25 molecules on T cells [49]. Furthermore, MSCs have also been shown to promote the generation and development of Tregs by secreting TGF-β. Of note, TGF-β is a two-edged sword in that it has immunosuppressive effects that alleviate liver inflammation [50] but can also promote the progression of liver fibrosis [51, 52].

In addition, MSCs are reported to exhibit immunomodulatory effects in macrophages, which play a central role in both fibrosis and fibrotic resolution in the liver. Watanabe et al. found that MSCs could change the polarity of macrophages toward an M2 anti-inflammatory phenotype, which involves the secretion of various anti-inflammatory factors, including chemokine ligand 1 (CCL-1) and IL-10, increases the production of matrix metalloproteinases to decrease ECM, and increases the phagocytosis of hepatocyte debris (during this process, macrophages increase the levels of pro-regenerative factors) [53]. Similar to the above study, murine adipose-derived MSCs were found to significantly increase the proportion of M2-like cells by increasing the production of IL-10 and arginase 1 activity [54]. In a mouse model of ischemia/reperfusion (IR)-induced sterile inflammatory injury of the liver, we found that adoptive transfer of MSCs reduced hepatocellular damage and shifted macrophage polarization from the M1 to M2 phenotype by increasing YAP and β-catenin nuclear translation in macrophages. Further research found that MSCs enhance the activity of the macrophage Hippo pathway, which in turn controls NLRP3 activation through a direct interaction between YAP and β-catenin and regulates XBP1-mediated NLRP3 activation, leading to reprogramming of macrophage polarization toward an anti-inflammatory M2 phenotype [55].

### Direct and indirect effects of MSCs on the fate of activated HSCs

Inflammation and fibrosis have a very close relationship in liver disease. When the liver is damaged, quiescent hepatic stellate cells (HSCs) transdifferentiate into proliferative myofibroblastic/activated HSCs, which are the main contributors to liver fibrosis. Investigators have tried to determine the methods by which MSCs may influence the fate of myofibroblasts/activated HSCs. Reported hypotheses include a direct effect by cell–cell contact and MSC-secreted cytokines/growth factors or an indirect effect by cellular mediators, such as macrophages or even hepatocytes. Regarding the indirect effects, MSCs can regulate the activities of HSCs by modulating immune cell activity. For example, MSCs can induce changes in the cytokine profile of activated macrophages by increasing the production of IL-10, PGE2 and matrix metalloproteinases and thereby promote the resolution of fibrosis [56]. On the other hand, MSCs can degrade ECM directly by secreting matrix metalloproteinases, such as MMP-13 and MMP-9 [57]. In vitro experiments have also demonstrated that after coculture with MSCs, the proliferation and activation of HSCs was inhibited by ADSCs, and the apoptosis of HSCs was promoted [58, 59]. Furthermore, a recent study reported that tumor necrosis factor-inducible gene 6 protein (TSG-6), a cytokine released from MSCs, could suppress HSC activation and induce the expression of stem cell markers in these cells. Then, the stem cell-like cells derived from HSCs treated with TSG-6 can form organoids that contribute to liver regeneration [60]. The milk fat globule-EGF factor 8 (MFGE8) was identified by Su et al*.* [61] as a novel key antifibrotic factor based on its role in the modulation of TGF-β signaling. In an in vivo analysis of mice, hUC-MSC secretomes were injected into mice with fibrosis, which led to a significant inhibition of liver fibrosis. MFGE8 is an antifibrotic protein in MSC secretomes that strongly inhibits TGF-β signaling and reduces extracellular matrix deposition and liver fibrosis in mice [62].

### Differentiation of MSCs into Hepatocyte-like Cells

A majority of in vitro studies demonstrated that MSCs had the capacity to differentiate into hepatocyte-like cells with liver-specific morphology and function with the help of specific growth factors, such as HGF, EGF, FGF, and OSM [63–66]. Furthermore, Yan et al*.* [67] showed that by mimicking the microenvironment of liver fibrosis using 50 g/L rat fibrotic liver tissue extracts, hUC-MSCs were stimulated to differentiate into hepatocyte-like cells in a shorter period of time. Similar results have been confirmed by in vivo experiments. Interestingly, Park et al*.* [68] previously transplanted human MSCs into rats with a fibrotic liver. Then, 21 days after transplantation, human albumin-positive cells were detected in the MSC infusion group, which suggested that the transplanted MSCs could differentiate into albumin-secreting hepatocyte-like cells in the damaged livers of the rats. In line with these findings, Zhang et al. [69] reported that the expression of human ALB, AFP, CK18 and CK19 were detected in the liver tissue of fibrotic and cirrhotic rats after hUC-MSC transplantation, suggesting that transplanted hUC-MSCs could migrate into the injured liver, where they could differentiate into hepatocyte-like cells. Furthermore, they also demonstrated that hUC-MSCs did not directly differentiate into functional hepatocytes; they first differentiated into epithelial cell-like cells and subsequently differentiated into hepatocyte-like cells. All the above findings indicated that MSCs could differentiate into hepatocyte-like cells through exposure to the liver fibrosis microenvironment both in vitro and in vivo. However, there are also many research reports that indicate that the trans-differentiation of MSCs into hepatocytes has rarely been observed (less than 1% of the total liver mass) in animal models after MSC infusion [70]. Similarly, menstrual blood-derived stem cells were demonstrated to improve liver function by eliminating collagen deposition and inhibiting proliferative HSCs via paracrine mediators, but few of the transplanted cells were found to differentiate into functional hepatocyte-like cells [71]. Based on the above studies, MSCs are believed to exhibit a positive treatment effect in two ways: directly by cell differentiation and indirectly by paracrine effects. Many studies have attempted to use exosomes or culture supernatants of MSCs to achieve therapeutic effects [72–74], indicating that the success of MSC therapy does not completely correlate with the efficiency of cell engraftment and replacement. For treatment, it is likely that the paracrine effects of MSCs play a more vital role. Furthermore, we believe that in some cases, alternative treatments, such as MSC exosomes or MSC culture supernatant therapy, may be more effective than treatment with MSCs themselves, as MSCs have robust plasticity, differentiation characteristics and immunomodulatory properties. Infusion of MSCs in different microenvironmental conditions may change their immunoregulatory characteristics, thereby affecting the stability of their therapeutic effects.

In conclusion, the treatment mechanisms mentioned above do not necessarily appear in the process of treating the same disease at the same time. According to the basic characteristics of different liver diseases, the role of MSCs in the treatment process may be focusing effect. For example, in applying for treatment inflammation related liver disease, such as hepatitis induced cirrhosis or complications of liver transplantation, the immunomodulatory function of MSCs may play a more vital role. However, for liver cirrhosis, the effects of MSCs on the fate of activated HSCs may also take part in the treatment process. In another hand, the efficacy of MSCs treatment in liver failure may mainly depend on its property of differentiation into hepatocyte-like cells.

## Modification of MSCs for therapy

Conventional unmanipulated MSCs have been the mainstay of therapeutic studies, and more efforts need to be made to enhance their efficacy. In fact, priming of MSCs before use is intended to enhance their biological properties and function in some animal models. This may involve the combination of MSCs with other factors, pretreatment and genetic modification of MSCs, and the use of secretomes of MSCs.

### Pretreatment of MSCs

Pretreatment of MSCs may improve their potential to treat liver diseases by increasing their homing, differentiation and immunoregulation capacity. Pretreatment of MSCs with zeaxanthin dipalmitate (ZD) could increase the cell survival rate and enhance the capacity of MSCs to differentiate into hepatocytes. In addition, ZD-pretreated MSCs ameliorated liver injury to a great extent [75]. The anti-inflammatory property of MSCs could be modulated by the inflammatory microenvironment. Thus, pretreatment with inflammatory factors such as TNF-α combined with IFN-γ may promote the anti-inflammatory ability of MSCs and shorten the functioning time in vivo [76]. However, the anti-inflammatory effect of MSCs is probably repressed after differentiation. Should we enhance the differentiation ability or immune regulation ability of MSCs by priming? This may depend on the specific disease type. In addition to MSC priming, the pretreatment of MSC recipients also exerted a similar effect in vivo. Pretreatment of recipients with both anti-PMN [77] and IL-1β siRNA [78] could promote the therapeutic efficacy of MSCs. Dexamethasone has been demonstrated to repress the repair capacity of MSCs [79]. Thus, dexamethasone should be withdrawn before MSC administration.

### Genetic modification of MSCs

In addition to MSC pretreatment, genetic modification of MSCs may be another powerful approach for disease therapy. To date, there have been many genetic modifications targeting MSC therapy in animal models. Both c-Met [80] and CXCR4 [81] overexpression could enhance the migration and engraftment of MSCs into injured sites and thus promote liver injury repair. In addition to showing the enhancement of homing, IGF-1-modified MSCs were found to have an enhanced anti-fibrotic ability compared to normal MSCs [82]. In fact, a large proportion of gene modifications of MSCs have been applied in liver cancer treatment. MSCs can deliver cytokines, such as IFN-β [83, 84], IFN-α2b [85], tumor necrosis factor-related apoptosis inducing ligand (TRAIL) [86, 87] and IL-12 [88], to inhibit hepatocarcinogenesis. MSCs also deliver the immune effector molecule CD3scfv to stimulate antitumor immunity [89]. In addition, MSCs can deliver the suicide gene HSV-Tk to induce cytotoxicity in hepatoma cells [90]. Overall, MSC-delivered cytokines could be more stable and longer lasting than pure cytokines and produce reduced side effects. Moreover, the use of genetically modified MSCs would be more safe than transfecting viruses directly into the body. However, considering the potential of MSCs to promote tumor growth, a safety evaluation should be carried out strictly before clinical application.

### Cell-free therapy with MSCs

The status of transplanted cells in vivo and the potential tumorigenic risks of MSCs have raised concerns about their effectiveness and safety. MSCs have been shown to exert their anti-inflammatory and tissue repair functions through the paracrine effects of cytokines and other secretomes. As a result, increasing evidence has confirmed the efficacy of conditioned medium (CM) and exosomes of MSCs in animal models of liver disease. MSC-CM exerts anti-inflammatory functions via production of anti-inflammatory cytokines, such as IL-10, IL-13, and IL-27 [91], and contributes to the expansion of immune-suppressive T cells (Tregs) [92]. MSC-CM also restored liver function by promoting hepatocyte proliferation and inhibiting hepatocyte apoptosis through HGF and VEGF secretion [93]. Moreover, Li et al*.* [72] found that UC-MSC-derived exosomes inhibited liver fibrosis by repressing EMT of hepatocytes and collagen production in a mouse model. Exosomes also inactivate the TGF-β1/Smad signaling pathway, which is involved in fibrogenesis. On the other hand, miRNAs in exosomes contribute to inhibiting fibrosis formation [94]. Thus, the use of the MSC secretome as an acellular therapeutic agent could provide several advantages over the use of cell-based therapies for liver diseases.

## Prospective of clinical application of MSCs

**Fig. 4:**
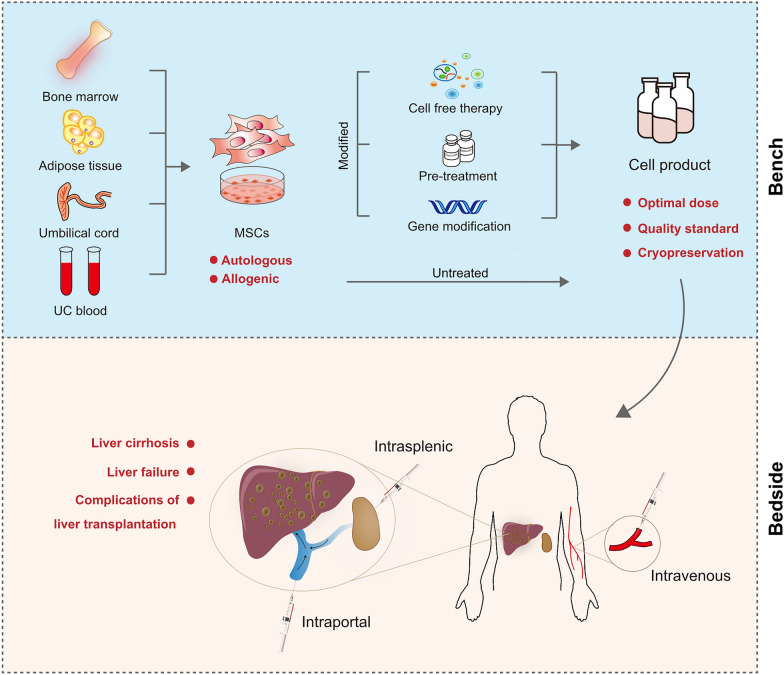
Concerns of MSC therapy for liver disease from bench to bedside

MSC therapy has been generally regarded as a safe and promising therapeutic strategy in clinical trials for patients with liver disease, including complications due to liver transplantation, liver failure, cirrhosis due to alcohol, HBV, or HCV, and primary biliary cholangitis. However, the application of MSCs for the treatment of related diseases has not been approved by official agencies such as the FDA, and this therapeutic strategy has not been as popular as expected. We speculate that this is probably because there are still some vital problems that have not been solved in the application of MSC therapy (Fig. 4). In addition, the inconsistency and ambiguity of MSCs as a type of stem cells may be one of the main reasons for the limited application of MSCs. The creator of the term "MSC", Arnold Caplan, recommended using "Medicinal Signaling Cells" instead of "Mesenchymal stem cells" to best describe these cells [95]. MSCs have been found as stem cells at first because of their self-renewal and multi-differentiation. However, their application will be greatly limited if we treat MSCs only as stem cells. MSCs also have some properties beyond stemness, such as migrating to injury sites and immunoregulation. These properties are important for liver disease therapy. Balancing different views of using MSCs as stem cells or other cell type will improve application of MSCs in liver disease therapy.

First, it is important to maintain the quality and stability of MSCs used for disease therapy. Based on the clinical trials mentioned above, there is no uniform standard for evaluating the quality of MSCs in each trial. The characteristics of MSCs from autologous bone marrow from different patients are likely to be quite different due to the diversity of factors such as patient age, gender, disease status, and other factors. For instance, a researcher in Brazil reported that MSCs isolated from multiple sclerosis patients have distinct gene expression profiles and functional defects implicated in MSC immunomodulatory and immunosuppressive activity compared with those from their healthy counterparts [96]. Another group also demonstrated that MSCs derived from hepatitis B patients proliferated slowly and tended to undergo senescence [97]. Therefore, we believe that the establishment of a complete MSC quality standard evaluation system is needed. Based on such a standard, we can know which MSCs from autologous patients are qualified or meet the standards and which should be eliminated and replaced with allogenic MSCs for treatment.

For the treatment of disease, the optimal dose of MSCs and the number of infusions is not well defined. To date, a very few articles have examined how many doses of MSCs could produce the best effect and whether transplanting more MSCs results in a better treatment effect. Therefore, we also have no idea what the optimal number of injections and interval times would be. By integrating data from the different clinical trials mentioned above, we believe that MSCs should be effective when the number of administered MSCs is within a certain range. Freshly isolated MSCs showed better homing than cultured cells [98]. Thus, the most suitable dose should be determined based on balancing the corresponding therapeutic effect and the time and economic costs required to obtain the corresponding dose of MSCs. Furthermore, as we mentioned in the third part of the review, it is not effective to perform multiple repeated MSC injections in a short period of time, such as within 6 months. In conclusion, low doses and multiple injections with longer intervals are probably the most effective and economical strategy for MSC application.

Judging from the large number of in vivo studies of MSCs, regardless of whether MSCs were obtained from autologous or allogenic sources or from bone marrow, umbilical cord or fat, MSC therapy did not cause serious adverse reactions, tumor formation or transplant-related deaths in clinical trials. Thus, the source of MSCs that should be used depends on the specific situation. For instance, allogeneic MSCs, compared with autologous MSCs, have more potential advantages for the treatment of ACLF. Because a characteristic of ACLF is its rapid progression, the use of allogeneic MSCs may be preferred because of their immediate availability, while it always needs to take longer time for culturing autologous MSCs to obtain a sufficient quantity.

Of note, another central issue for the future success of cell transplantation is the ability to noninvasively assess the fate, migration patterns, and survival of transplanted cells. If we can track the fate of the transplanted MSCs, we could know the proportion of cells that home in and engraft in the liver and the way that cells exert their therapeutic effects. The classic method of MSCs labelling uses retroviral vectors to express fluorescent protein or gene-modified animal models to track MSCs in vivo [99, 100]. However, the visualization of cells that home in in different organs requires sacrificing of the animal, as the tissue penetrability of fluorescence is limited. Therefore, these technologies are not suitable for clinical applications. More noninvasive techniques have been employed constantly. Endomicroscopy was reported as a clinically available noninvasive cellular tracking method used to track cell fate in vivo [101]. Furthermore, cellular magnetic resonance imaging (MRI) using fluorine- or iron-based nanoemulsions is also regarded as a great means to detect transplanted cells in vivo because of its high specificity and precise quantification [102, 103]. However, the potential limitations of these tracking tools are the possibility of transferring the label to local bystander cells and the effect of the label on MSC basic feature. More advanced and improved technology for transplanted cell tracking needs to be employed.

## Conclusions

According to clinical trials, MSC therapy has been regarded as a safe and promising therapeutic strategy for patients with end-stage liver disease, including liver cirrhosis, liver failure, and complications after liver transplantation. However, for MSCs to become a routine treatment for these liver diseases and to be widely used, further studies with larger samples and double-blind controlled trials are needed to increase the safety and efficacy of MSCs in the clinic. Furthermore, studies on the optimal MSC transplantation route, minimum effective number of MSCs, and tracking of MSCs are needed to provide more references and guidance for specific future clinical treatment programs. In addition to the use of conventional unmanipulated MSCs, derived MSC therapies, such as the use of primed MSCs or gene-modified MSCs or cell-free therapy, may be promising and practical treatments in the future.

## Data Availability

Not applicable.

## Abbreviations

MSCs: Mesenchymal stem cells
LC: Liver cirrhosis
MELD score: Model for end-stage liver disease scores
ACLF: Acute-on-chronic liver failure
GCSF: Granulocyte colony stimulating factor
BM: Bone marrow
ALF: Acute liver failure
UC: Umbilical cord
AD: Adipose tissue
IL-17: Interleukin-17
TNF-α: Tumor necrosis factor-α
IL-6: Interleukin-6
iNOS: Inducible nitric oxide synthase
HGF: Hepatocyte growth factor
PGE2: Prostaglandin E2
TGF: Transforming growth factor
MMPs: Matrix metalloproteinases
CCL-1: Chemokine ligand 1
IR: Ischemia/reperfusion
HSCs: Hepatic stellate cells
TSG-6: Tumor necrosis factor-inducible gene 6 protein
MFGE8: Milk fat globule-EGF factor 8
ZD: Zeaxanthin dipalmitate
TRAIL: Tumor necrosis factor-related apoptosis inducing ligand
CM: Conditioned medium
